# Psychological Burden of the COVID-19 Pandemic on Healthcare Workers: A Comparative Study Across Sectors and Clinical Roles

**DOI:** 10.7759/cureus.91426

**Published:** 2025-09-01

**Authors:** Jagadeesan Subramanian, Ramakrishnan T Venkatakrishnan, Srihari Cattamanchi, Kennedy Kumar, Sugandhi Pugazhendhi, Krishna Kumar Dharuman

**Affiliations:** 1 Department of Emergency Medicine, Sri Ramachandra Institute of Higher Education and Research (Deemed to be University), Chennai, IND; 2 Department of Microbiology, Sri Ramachandra Institute of Higher Education and Research (Deemed to be University), Chennai, IND; 3 Medical and Bioresearch, Aarupadai Veedu Medical College and Hospital, Vinayaka Mission's Research Foundation (Deemed to be University), Puducherry, IND; 4 Department of Emergency Medicine, Institute of Allied Health Science, Srinivas University, Mangalore, IND

**Keywords:** anxiety, coronavirus disease 2019, covid-19 pandemic, depression, frontline health workers, government hospitals, hamilton anxiety scale, hamilton rating scale for depression, private hospitals, stress

## Abstract

Background and aim: The COVID-19 pandemic has impacted healthcare workers' mental health, causing stress, anxiety, burnout, and post-traumatic stress disorder. This study evaluated psychological indicators across government and private healthcare settings to develop targeted mental health interventions for healthcare workers.

Methodology: This cross-sectional study was conducted over one year at the Sri Ramachandra Institute of Higher Education and Research, Chennai, India, and the Government Headquarters Hospital in Kanchipuram, India. The study involved 400 healthcare workers from each tertiary care hospital. A stratified random sampling method categorized workers into frontline and non-frontline groups. Data were collected using a custom questionnaire for stress and demographic data, and standardized questionnaires for anxiety and depression: the Hamilton Anxiety Rating Scale (HAM-A) and Hamilton Rating Scale for Depression (HAM-D). Statistical analyses used independent t-tests and chi-square tests.

Results: Frontline workers showed elevated incidences of severe anxiety (n=22, 22%) and depression (n=32, 31.8%) compared to non-frontline counterparts. Non-frontline workers reported higher levels of mild-to-moderate stress and anxiety. Healthcare professionals in the private sector showed higher levels of severe stress (n=78, 39%), anxiety (n=88, 44%), and depression (n=84, 42%) than those in the government sector. Significant differences were observed in psychological assessments between frontline and non-frontline workers and between government and private-sector workers.

Conclusion: This study shows the impact of the COVID-19 pandemic on healthcare workers' psychological well-being. Frontline workers faced higher rates of severe anxiety and depression, while non-frontline workers experienced notable levels of mild-to-moderate stress. These findings emphasize the need for support systems and interventions tailored to address the needs of frontline and non-frontline healthcare workers across government and private sectors.

## Introduction

The COVID-19 pandemic has not only strained healthcare systems globally but has also had a significant impact on the mental health of healthcare workers (HCWs). The psychological impact of these frontline professionals extends beyond immediate stress and anxiety associated with crisis management [1]. Long-term effects include burnout, compassion fatigue, and post-traumatic stress disorder (PTSD), which can persist long after the acute phase of the pandemic [2]. 

The constant fear of contracting the virus and potentially transmitting it to loved ones adds another layer of emotional burden, leading to social isolation and strained personal relationships. Moreover, the pandemic has highlighted existing systemic issues in healthcare, such as understaffing, inadequate resources, and insufficient mental health support for HCWs. The overwhelming workload, coupled with ethical dilemmas in resource allocation and end-of-life care decisions, has forced many HCWs to confront moral distress and question their professional identity [3]. This complex interplay of factors has underscored the urgent need for comprehensive mental health interventions tailored to the unique needs of healthcare professionals as well as systemic changes to create more resilient and supportive healthcare environments in preparation for future crises [4].

This study focuses on evaluating these mental health indicators across both government and private healthcare settings, which is crucial for developing an understanding of how different work environments influence the psychological impact on HCWs. This study sought to provide valuable insights into the psychological well-being of HCWs during the pandemic, potentially informing strategies to support and protect this crucial workforce in both the government and private healthcare sectors.

The potential of this research to inform evidence-based interventions and support systems is particularly significant. By identifying specific psychological challenges faced by HCWs in various healthcare settings, this study can guide the development of targeted mental health support programs ranging from on-site counselling services and stress management workshops to the implementation of more flexible work schedules and improved safety protocols. Furthermore, the findings could influence policy decisions at both the institutional and governmental levels, potentially leading to the allocation of more resources for mental health support in healthcare settings. This comprehensive approach to understanding and addressing the psychological impact on HCWs is essential not only for their well-being but also for maintaining the overall resilience and effectiveness of healthcare systems in the face of ongoing and future health crises.

## Materials and methods

This was a cross-sectional study conducted at Sri Ramachandra Institute of Higher Education and Research (Deemed to be University), Chennai, India, and Government Headquarters Hospital, Kanchipuram, India (COVID Care Centers), from May 1, 2022, to April 30, 2023. The study was approved by the Institutional Ethics Committee, Sri Ramachandra Institute of Higher Education and Research (reference number: IEC-NI/22/JAN/81/29). The study was conducted under the Indian Council of Medical Research's updated National Ethical Guidelines for Biomedical and Health Research Involving Human Participants, 2017.

Eligibility criteria

HCWs who were working at both the Sri Ramachandra Institute of Higher Education and Research and the Government Headquarters Hospital were included in the study. Eligible participants were HCWs working in COVID care facilities, aged 18-65 years, with a minimum of three months of pandemic experience. Eligibility extended to both frontline workers and non-frontline staff members who provided informed consent for participation. Exclusion criteria included HCWs with less than three months of COVID care experience, those unwilling to provide consent, and pregnant healthcare workers. HCWs with pre-pandemic mental health disorders or those using psychotropic medications were also excluded from the psychological assessment.

In this study, a frontline HCW was defined as an HCW who was involved in the diagnosis, treatment, or nursing care of COVID-19 patients (nurses, paramedics, doctors). HCWs who had no contact with the units assigned to handle services for COVID-19 patients were defined as non-frontline HCWs (housekeeping, security, supervisor, administrative officer, lab technician).

Sample size

According to an earlier study by Al Maqbali et al., the prevalence of stress, anxiety, depression, and sleep disturbance was 37%, 31.8%, 29.4% and 36.9%, respectively [5]. Assuming that 35% of the HCWs would have depression or anxiety, it was calculated that the study would require a sample size of 350 from each tertiary care hospital to estimate the expected proportion with 5% absolute precision at a 95% confidence level (CI).

Sampling method

A stratified random sampling method was employed, classifying workers as either frontline or non-frontline. Separate lists of frontline and non-frontline workers were generated from each tertiary care facility. A random list comprising 125 workers from each group was developed, and after adjusting for the eligibility criteria, 100 HCWs were included in the study from each category from the two study centers.

Data collection for stress and demographic details

Stress levels were evaluated through a custom semi-structured questionnaire (see Appendices). The questionnaire underwent pretesting and was appropriately modified. It is noteworthy that no separate validated stress-specific scale was employed for this assessment. Participants self-assessed their stress as mild, moderate, or severe, based on their perceived work pressure, emotional strain, and fatigue experienced during the COVID-19 pandemic.

Data was also collected on demographic information (gender, age, BMI, education), participants’ job roles, and their involvement in clinical activities, such as diagnosing, treating, or delivering nursing care to patients with confirmed COVID-19 who exhibit elevated temperatures. Finally, participants were asked if they had previously sought help for psychiatric disorders (‘yes’ / ‘no’).

Assessment tools for anxiety and depression

The Hamilton Anxiety Rating Scale (HAM-A) [6] and Hamilton Depression Rating Scale (HAM-D) [7], originally developed by Hamilton in 1959 and 1960, respectively, were utilized for evaluating anxiety and depression. These instruments are available for public use. The HAM-A and the HAM-D are the first rating scales created to assess the intensity of symptoms related to anxiety and depression and are widely utilized in both clinical and research studies.

The HAM-A

The HAM-A was specifically developed to evaluate the seriousness of anxiety symptoms. The scale ranges from 0 to 4, with 0 indicating no anxiety symptoms present and 4 signifying very severe symptoms. It consists of 14 items, including anxious mood, tension, fears, and disturbances in sleep, cognitive issues, depressed mood, musculoskeletal complaints, sensory issues, cardiovascular symptoms, respiratory problems, gastrointestinal disturbances, genitourinary concerns, autonomic nervous system symptoms, and behavior during the interview. An overall anxiety level was represented by a total score, which was determined by summing up the scores assigned to each of the individual items.

The HAM-D

The HAM-D was formulated to assess the intensity of depression symptoms. A clinician evaluates the participant based on 17 items, which were scored using either a three-point or a five-point Likert-type scale. This scale features 17 items, including Depressed Mood, Feelings of Guilt, Suicidal Thoughts, Initial Insomnia, Middle Insomnia, Late Insomnia, Work and Activities, Retardation, Agitation, Psychic Anxiety, Somatic Anxiety, Gastrointestinal Somatic Symptoms, General Somatic Symptoms, Genital Issues, Hypochondriasis, Loss of Weight, and Insight. The overall score, calculated by adding the points assigned to each item, indicates the overall level of anxiety. A score ranging from 0 to 7 was considered normal, while a score of 20 or greater, indicating at least moderate severity, was typically necessary for participation in a clinical trial.

Statistical analysis

Demographic information was analyzed using independent t-tests. The prevalence of depression, anxiety, and stress among HCWs was assessed with 95% confidence intervals (CIs) for each category in both tertiary care hospitals. The chi-squared test was employed to compare various proportions, with a p-value of less than 0.05 deemed statistically significant. All analyses were performed with IBM SPSS Statistics for Windows, version 25 (IBM Corp., Armonk, N.Y., USA).

## Results

The number of frontline HCWs was nearly equal between government (n=100; 50%) and private (n=98; 49%) centers. The difference was only 1% lower in private centers. There was a slight increase (1%) in non-frontline HCWs in private centers (n=102; 51%) compared to government centers (n=100; 50%). This suggests a marginally higher allocation of non-frontline workers in private healthcare facilities. Private centers might be allocating more staff to administrative or supportive roles than government centers. The almost equal frontline worker distribution suggested that both types of centers maintain similar staffing levels for direct patient care (Table 1).

**Table 1:** Distribution of frontline and non-frontline healthcare workers in government and private tertiary care hospitals (N=400). HCW: healthcare worker

HCW Category	Government Hospitals, n (%)	Private Hospitals, n (%)	Difference
Frontline HCWs	100 (50%)	98 (49%)	1% lower (Private)
Non-Frontline HCWs	100 (50%)	102 (51%)	1% higher (Private)
TOTAL	200	200	400

Demographic details of frontline and non-frontline HCWs

The average age of frontline HCWs was 36.98 years (±6.9), while non-frontline HCWs had a slightly higher average age of 38.86 years (±8.6). Among front-line workers, 115 (58%) were aged 16-30 years, whereas only 45 (22%) non-front-line workers fell into this age group. In terms of gender distribution, 118 (29.5%) frontline HCWs were male, compared to 155 (38%) in the non-frontline group. Females comprised 282 (70.5%) of frontline workers and 253 (62%) non-frontline workers. Regarding BMI, 100 (50%) frontline HCWs fell within the 19.6-24.9 kg/m² range, and 110 (54%) non-frontline workers were in the same category. Occupation-wise, 124 (62%) frontline HCWs were female nurses, 62 (31%) were technologists, and smaller percentages were paramedics, doctors, male nurses, and other roles. Non-frontline workers included 74 (37%) from the administrative office, 59 (29%) from security, 40 (20%) from housekeeping, and smaller proportions from other fields. Education levels showed that 127 (64%) and 130 (64%) HCWs of each group were graduates, but postgraduates were significantly more common among frontline workers (n=71; 36%) than non-frontline workers (n=19; 10%).

The comparison between frontline and non-frontline HCWs based on the demographic variables shows statistical significance in some areas but not in others. The p-values indicate whether the differences observed between the two groups were statistically significant. The only statistically significant difference (p = 0.000) was in occupation, meaning frontline and non-frontline HCWs had distinct job roles. Other factors like age, gender, BMI, marital status, and family type showed no significant differences (p > 0.05), suggesting these variations occurred by chance rather than due to a true underlying difference (Table 2).

**Table 2:** Demographic characteristics of frontline and non-frontline healthcare workers (N=400) *Chi-square test Data given as n (%), except for age, which is given as mean ± SD BMI: body mass index; SD: standard deviation

Demographic variable	Category	Frontline HCWs (n=198), n (%)	Non-frontline HCWs (n=202), n (%)	p-value*
Age (years)	mean ± SD	36.98 ± 6.9	38.86 ± 8.6	0.117
Age group	16–30	115 (58%)	45 (22%)	0.124
	31–45	83 (42%)	104 (51%)	
	46–60	–	51 (25%)	
	61–75	–	2 (2%)	
Gender	Male	118 (29.5%)	155 (38%)	0.000
	Female	282 (70.5%)	253 (62%)	
BMI (kg/m²)	18.5–19.5	49 (25%)	50 (26%)	0.118
	19.6–24.9	100 (50%)	110 (54%)	
	25–26.5	49 (25%)	42 (20%)	
Occupation	Female nurse	124 (62%)	–	0.000*
	Technologist	62 (31%)	–	
	Paramedic	3 (1.5%)	–	
	Doctor	6 (4%)	–	
	Male nurse	3 (1.5%)	–	
	Security	–	59 (29%)	
	Admin office	–	74 (37%)	
	Driver	–	16 (8%)	
	Housekeeping	–	40 (20%)	
	HWT	–	13 (6%)	
Education	High school	–	53 (26%)	0.214
	Graduate	127 (64%)	130 (64%)	
	Postgraduate	71 (36%)	19 (10%)	

Psychological assessment of stress in frontline and non-frontline HCWs

**Figure 1:**
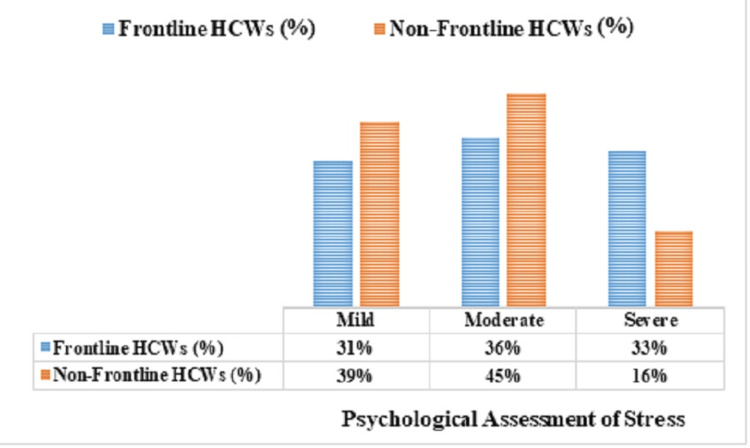
Psychological assessment of stress levels among frontline and non-frontline HCWs during the COVID-19 pandemic (N = 400) HCW: healthcare workers

A higher proportion of non-frontline HCWs experienced mild (n=79, 39%) and moderate (n=91, 45%) stress compared to frontline workers (n=61 (31%) and n=71 (36%), respectively). Non-frontline HCWs report higher levels of mild (39% vs. 31%) and moderate (45% vs. 36%) stress compared to frontline workers. However, severe stress was more prevalent among frontline workers (n=65, 33%) than non-frontline workers (n=32, 16%) (χ² = 9.861, p = 0.005, 95%CI: 1.321 -3.423) (Figure 1). There was a statistically significant difference in stress levels between frontline and non-frontline HCWs.

Psychological assessment of anxiety in frontline and non-frontline HCWs

**Figure 2:**
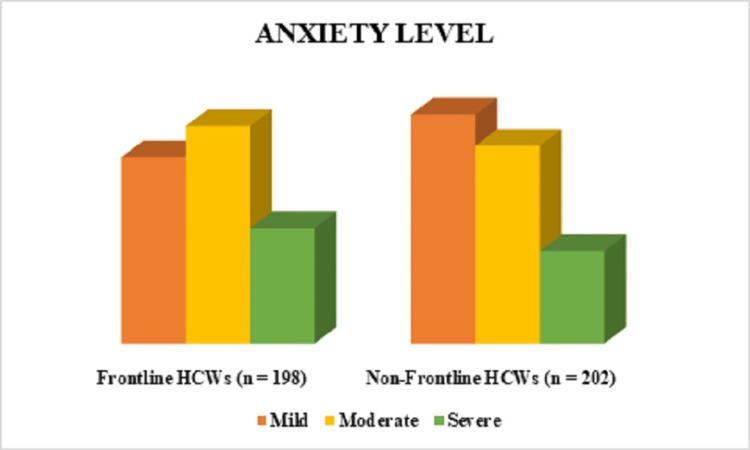
Psychological assessment of anxiety levels among frontline and non-frontline HCWs during the COVID-19 pandemic (N=400) HCW: healthcare worker

The results of anxiety showed that among frontline HCWs, 71 (36%) experienced mild anxiety, 83 (42%) had moderate anxiety, and 44 (22%) suffered from severe anxiety. In contrast, among non-frontline HCWs, 89 (44%) reported mild anxiety, 77 (38%) experienced moderate anxiety, and 36 (18%) had severe anxiety. To statistically interpret the differences in anxiety levels between frontline and non-frontline HCWs, a chi-square test for independence was used to determine whether there was a significant association between frontline vs. non-frontline and anxiety severity (mild, moderate, severe). The chi-square value was on the borderline of statistical significance (χ² = 8.996, p = 0.005, 95%CI: 1.222 - 2.423). Based on these data, the results concluded that frontline HCWs experienced higher levels of severe anxiety compared to non-frontline workers, but the difference was only marginally significant. Non-frontline HCWs reported slightly higher mild anxiety than frontline workers. The moderate anxiety levels were similar between the two groups (Figure 2). The chi-square test suggested a borderline statistically significant relationship between the frontline and non-frontline HCWs. 

Psychological assessment of depression in frontline and non-frontline HCWs

**Figure 3:**
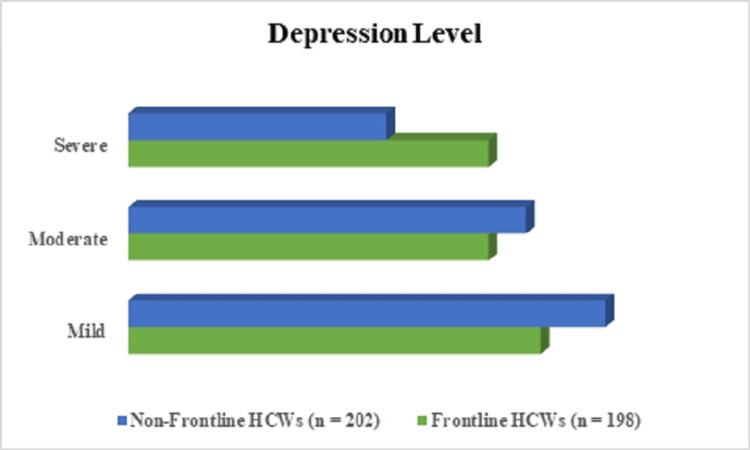
Psychological assessment of depression levels among frontline and non-frontline HCWs during the COVID-19 pandemic (N=400) HCW: healthcare worker

The results of depression indicated that among frontline HCWs, 72 (36.4%) experienced mild depression, 63 (31.8%) had moderate depression, and 63 (31.8%) suffered from severe depression. In contrast, among non-frontline HCWs, 85 (42%) had mild depression, 71 (35%) experienced moderate depression, and 46 (22.8%) suffered from severe depression (Figure 3). Since the p-value (0.02) was less than 0.05, this means there was a statistically significant association (χ² = 11.21, p = 0.02, 95%CI: 2.333 - 4.214) between HCW type (frontline vs. non-frontline) and depression severity. In other words, the distribution of depression levels differed significantly between the two groups.

Psychological assessment of HCWs in the government sector

The assessment of psychological conditions among the surveyed individuals in the government sector revealed varying degrees of stress, anxiety, and depression. Stress levels were categorized as mild in 62 (31%) participants, moderate in 66 (33%), and severe in 72 (36%). Anxiety was reported as mild in 44 (22%), moderate in 84 (42%), and severe in 72 (36%) respondents. Similarly, depression was found to be mild in 63 (31.5%), moderate in 64 (32%), and severe in 73 (36.5%) individuals (Table 3). There was a high prevalence of psychological burden; in all three domains, stress, anxiety, and depression, the majority of government HCWs experienced moderate to severe symptoms. Anxiety shows statistical significance (p = 0.002), indicating a notable difference, making it an area of concern. Stress and depression do not show significant differences statistically, though the levels are still high and require attention.

**Table 3:** Psychological assessment of stress, anxiety, and depression among government HCWs (n = 200) *Chi-square test HCW: healthcare worker

Domain	Level	Government HCWs (n = 200), n (%)	p - value*
Stress	Mild	62 (31%)	0.437
	Moderate	66 (33%)	
	Severe	72 (36%)	
Anxiety	Mild	44 (22%)	0.002
	Moderate	84 (42%)	
	Severe	72 (36%)	
Depression	Mild	63 (31.5%)	0.402
	Moderate	64 (32%)	
	Severe	73 (36.5%)	

Psychological assessment of HCWs in the private sector

Psychological assessment of examined participants from the private sector revealed notable severity levels across various domains. Thirty-two (16%) HCWs experienced mild stress, 90 (45%) suffered from moderate stress, and 78 (39%) endured severe stress. Similarly, anxiety levels were well distributed with 36 (18%) at a mild level, 76 (38%) at a moderate level, and 88 (44%) at a severe level. Depression was reported as mild by 46 (23%) participants, moderate by 70 (35%), and severe by 84 (42%) (Table 4). Across all three domains, stress, anxiety, and depression, a large majority of private HCWs experienced moderate to severe symptoms. All three domains show highly significant p-values, indicating a real psychological burden that differs significantly from that of the government HCWs. This shows that HCWs employed in private hospitals may have faced greater psychological distress, possibly due to factors like job insecurity and longer working hours.

**Table 4:** Psychological assessment of stress, anxiety, and depression among private HCWs (n = 200) *Chi-square HCW: healthcare worker

Domain	Level	Private HCWs (n = 200), n (%)	p - value*
Stress	Mild	32 (16%)	0.003
	Moderate	90 (45%)	
	Severe	78 (39%)	
Anxiety	Mild	36 (18%)	0.000
	Moderate	76 (38%)	
	Severe	88 (44%)	
Depression	Mild	46 (23%)	0.002
	Moderate	70 (35%)	
	Severe	84 (42%)	

## Discussion

This study's findings highlight the significant psychological impact of the COVID-19 pandemic on HCWs in both the government and private sectors. The results reveal a complex pattern of stress, anxiety, and depression among frontline and non-frontline workers, emphasizing the need for tailored mental health interventions.

Frontline workers have been found to experience elevated rates of severe anxiety (n=44, 22%) and depression (n=63, 31.8%) compared to their non-frontline counterparts. This observation is consistent with prior research that highlights the increased psychological burden faced by individuals directly engaged in the care of COVID-19 patients [8,9]. Factors such as continuous exposure to the virus, fear of transmission, and ethical challenges in resource allocation are likely to contribute to this heightened stress [10]. Cai et al. conducted a study involving 1,173 frontline medical workers and 1,173 matched non-frontline medical workers, revealing that frontline staff exhibited significantly higher rates of mental health issues, anxiety, depressive symptoms, and insomnia [11]. Similarly, Tong et al. analyzed approximately 19 studies and found that frontline HCWs demonstrated pooled prevalence rates of anxiety (43%) and depression (44.6%), which were substantially higher than the averages observed among general HCWs [12]. Conversely, Elbay et al. identified elevated stress levels among non-frontline physicians compared with certain frontline groups, attributing this to job role ambiguity and diminished access to institutional support [13]. Spoorthy et al. identified that HCWs experienced significant psychological distress, particularly manifesting as stress, anxiety, depressive symptoms, and insomnia, during the initial phase of the pandemic [14]. Collectively, these comparisons suggest that, while our findings are consistent with global trends regarding the vulnerability of frontline workers, stressors specific to certain sectors may further impact psychological outcomes.

Notably, non-frontline HCWs reported elevated levels of mild-to-moderate stress and anxiety, indicating that the psychological impact of the pandemic extended beyond those directly involved in patient care, affecting the entire healthcare system. In this study, mild and moderate stress were reported by 79 (39%) and 91 (45%) non-frontline HCWs, respectively, compared to 61 (31%) and 71 (36%) frontline HCWs. Contributing factors may include alterations in work routines, job insecurity, and pervasive crises [15]. A survey of 467 HCWs, comprising 244 frontline and 223 non-frontline staff members, identified significantly higher incidences of depression (27% vs. 11%), anxiety (27% vs. 14%), and stress (15% vs. 8%) among non-frontline personnel [16]. This disparity was attributed to perceptions of inadequate PPE, insufficient institutional support, discrimination, and limited training, which were more prevalent among non-frontline staff. A comparative study involving 524 medical staff indicated mild-to-moderate anxiety and depression across both groups, with elevated occupational stress strongly correlated with mental health symptoms in both frontline and non-frontline workers [17]. The observed increase in mild-to-moderate stress and anxiety among non-frontline HCWs is consistent with national estimates from India. A meta-analysis conducted in the early stages of the COVID-19 pandemic reported pooled prevalence rates of 20.1% for depression, 25.0% for anxiety, and 36% for stress [18]. This level of psychological distress was similarly reflected in a mixed-methods review, which documented high prevalence rates of depression (32.96%), anxiety (29.49%), and stress (33.47%) among Indian HCWs [19]

The study identified significant differences between HCWs in the government and private sectors. Workers in the private sector exhibited higher levels of severe stress (n=78, 39%), anxiety (n=88, 44%), and depression (n=84, 42%) compared to their government counterparts (n=72, 36%; n=72, 36%; and n=73, 36.5%, respectively). This disparity may be attributed to factors such as job security, work hours, and resource availability, which vary between sectors. A large-scale study found that public-sector HCWs reported slightly higher anxiety (~25%) compared to their private-sector counterparts (~20%), although both groups demonstrated elevated stress levels overall. The public-private differential has been ascribed to disparities in resource allocation policies and institutional support [20]. These findings highlight the need for mental health support and improved safety protocols in healthcare settings. The study emphasizes addressing systemic issues, such as understaffing, that contribute to HCWs' psychological distress. The distribution of workers between government and private centers shows similar staffing levels, although private centers have more non-frontline administrative staff. While the study examined multiple psychological indicators, its limitations include its cross-sectional design and self-reporting bias. Future research should assess the long-term psychological impact and intervention effectiveness. This study provides insights into HCWs' psychological well-being during the COVID-19 pandemic, emphasizing the need for mental health support and systemic changes in healthcare resilience. Policies for the frontline should focus on shift limits, stable contracts, and emotional support, while non-frontline employees need flexible scheduling and mental health resources. Tailored interventions, informed by worker feedback, can reduce burnout and improve retention for both groups.

Limitations

As a cross-sectional study, this research captured the psychological status of HCWs solely during the study period, thereby precluding the establishment of temporal or causal relationships. Stress was assessed using custom items within a self-reported, semi-structured questionnaire rather than a standardized stress scale. Additionally, self-reported responses are susceptible to recall and reporting bias. Also, the findings of this study were derived from two tertiary care hospitals in Tamil Nadu, India, which may not comprehensively represent the experiences of HCWs in different geographic or healthcare contexts.

## Conclusions

This study assessed levels of stress, anxiety, and depression among frontline and non-frontline HCWs during the COVID-19 pandemic using the HAM-A, HAM-D, and a custom stress questionnaire. The findings indicated that frontline workers exhibited a higher incidence of severe anxiety and depression, whereas non-frontline workers reported mild-to-moderate stress levels. These results highlight the variations in psychological well-being based on occupational role and workplace sector, emphasizing the necessity for context-specific mental health support for healthcare professionals.

## Figures and Tables

**Figure 4:**
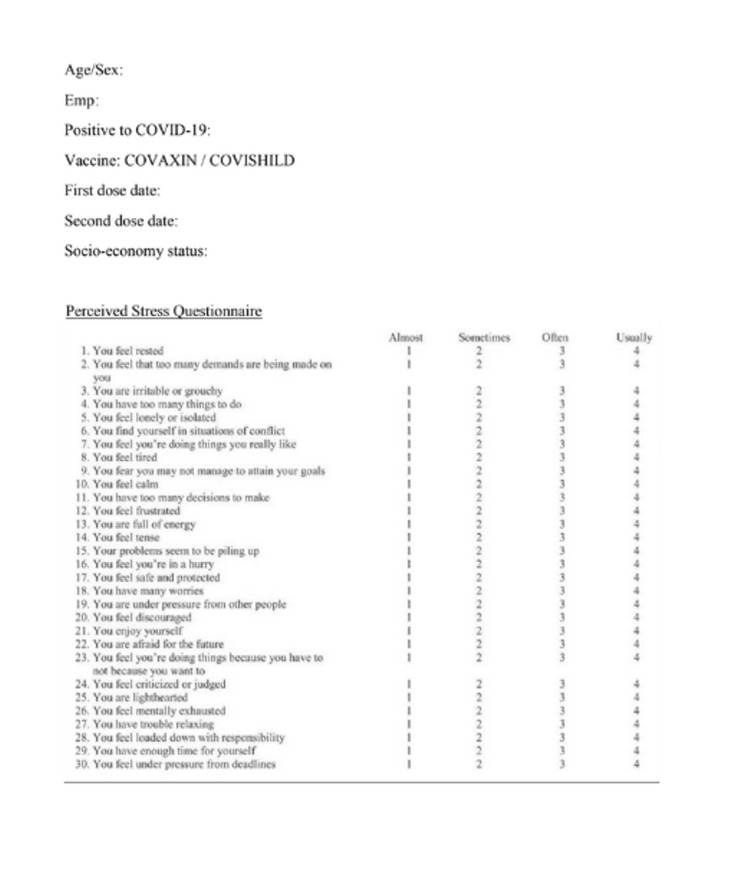
Custom questionnaire on stress and some demographic data

## Appendices

Study questionnaire on stress
